# Supplementary material for: Early stage prion assembly involves two subpopulations with different quaternary structures and a secondary templating pathway
Source: Commun Biol. 2019 Oct 4;2:363. doi: 10.1038/s42003-019-0608-y (PMC6778151; doi:10.1038/s42003-019-0608-y)
Supplement: Supplementary file 1 — Supplementary Information [file 42003_2019_608_MOESM1_ESM.pdf]

Supplementary Figures

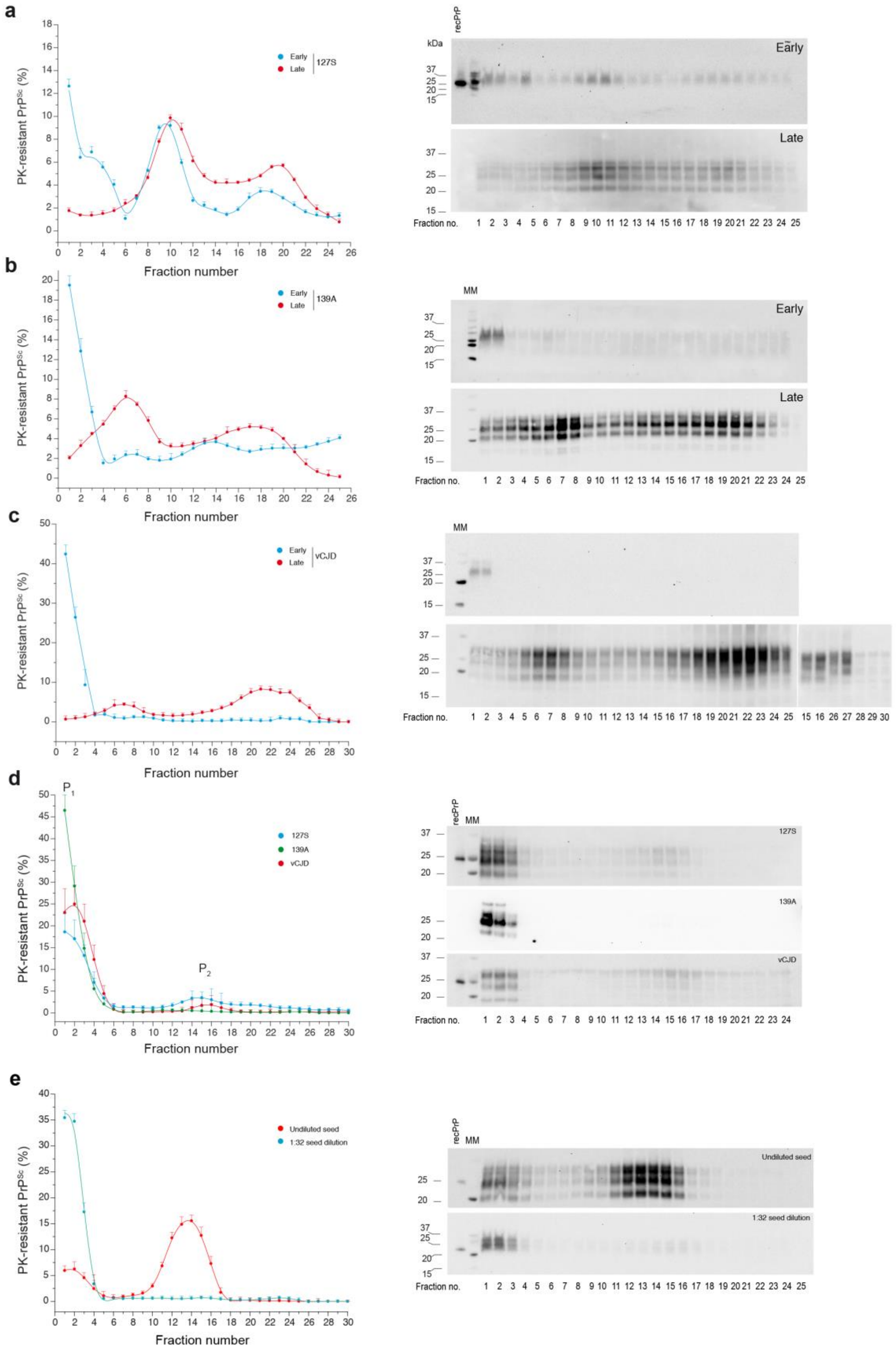

Supplementary Figure 1. Size distribution of PrP^{Sc} assemblies from different prion

strains at the early and late stages of pathogenesis *in vivo* and after the PMCA reaction

(**a-c**) For the *in vivo* sedimentograms, brains from ovine (tg338), murine (tga20) and human (tg650) transgenic mice inoculated with 127S scrapie prions (**a**), 139A mouse prions (**b**) and vCJD human prions (**c**) were collected (in triplicate) at the early stage (15 days postinfection (127S), 11 days postinfection (139A) and 120 days postinfection (vCJD), blue curves) and at the end stage of the disease (60 days postinfection (127S), 55 days postinfection (139A), 495 days postinfection (vCJD), red curves). The brains were solubilized and SV-fractionated. The collected fractions (numbered from top to bottom) were analyzed for PK-resistant PrP^{Sc} content by immunoblotting (mean ± SEM values obtained from *n=3* independent fractionations). Representative immunoblots at early and late stage are shown (right panel). Note that early brain PrP^{Sc} was electrophoretically concentrated on loading, so as to obtain signals amenable to relative quantification, hence the unusual, condensed PrP^{res} profile. Note that in panel **c**, the entire gradient was analysed because of the presence of higher-size vCJD PrP^{res} assemblies at late stage. Fractions 15 and 16 were loaded on the two gels to ensure consistent quantification among the gels. Molecular mass markers (MM) and recombinant PrP (recPrP) were loaded as controls.

(**d-e**) For the sedimentograms from the PMCA products with PrP^C substrate (**d**, left panel), the same prion strains were subjected to a single round of mb-PMCA by using 10^{-5} (139A) or 10^{-6} (vCJD, 127S) diluted brain homogenates as seed for the reaction. Thirty minutes after the last sonication, the amplified products were solubilized and SV-fractionated. The mean ± SEM levels of PK-resistant PrP^{Sc} per fraction were obtained from the immunoblot analysis of *n=4* independent fractionations of PMCA reactions. The peaks containing PrP^{Sc} assemblies sedimentating in the top and middle fractions were termed P_1 and P_2, respectively. Representative immunoblots are shown on the right panel. For the sedimentograms from the PMCA products without PrP^C substrate (**e**, left panel), undiluted 127S-infected tg338 brain

(20% w/v, red curve) or a 1:32 dilution in PMCA buffer (blue curve) was used as seed, mixed with brain homogenate from $PrP^{0/0}$ mice as substrate and subjected to a single round of mb-PMCA before SV fractionation (mean ± SEM levels from $n=3$ independent fractionations). Representative immunoblots are shown on the right panel. Note that PrP^{Sc} from the PMCA product seeded with 1:32 seed dilution was electrophoretically concentrated on loading, so as to obtain detectable and quantifiable signals, hence the unusual, condensed PrP^{res} profile.

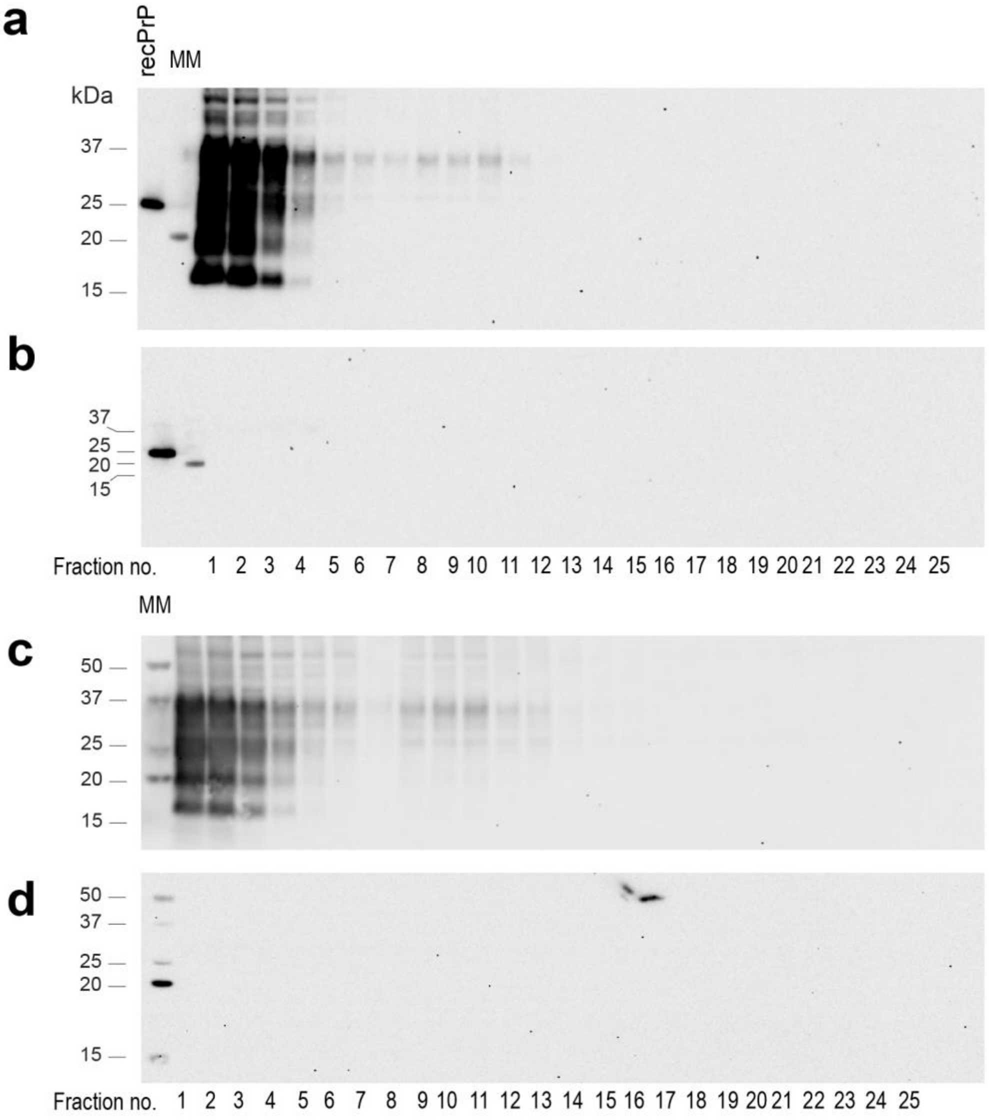

Supplementary Figure 2. Sedimentation velocity profile of PrP^C from uninfected tg338 mouse brain, submitted or not to PMCA

Brain homogenate from uninfected tg338 mice was used either crude (**a-b**) or as seed (10^{-3} dilution) in the mb-PMCA reaction (**c-d**). The brain homogenate (**a-b**) and the resulting PMCA product (**c-d**) were then solubilized and SV-fractionated. The collected fractions were analyzed for PrP^C content (**a, c**) before and (**b, d**) after PK treatment (80 µg/ml final concentration, 1h, 37°C). The gels are purposely overexposed to show the absence of residual,

PK-resistant PrP^{C} in the top fractions. The method applied to concentrate electrophoretically PrP^{Sc} in early brains (Fig. 1 & Supplementary Fig. 1) was used in panel b. Detection was made with Sha31 anti-PrP antibody. Recombinant ovine PrP (recPrP) was used as loading control in panels **a** and **b**. MM: molecular mass markers.

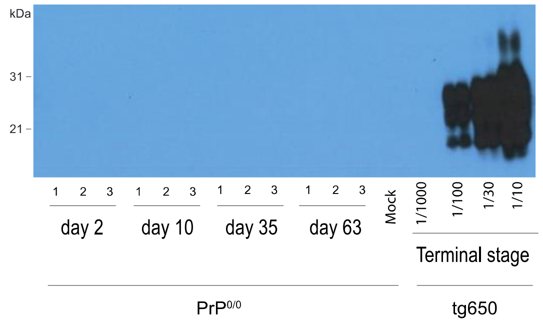

Supplementary Figure 3. Absence of detection of PrP^{res} in the brains of PrP$^{0/0}$ mice inoculated intracerebrally with vCJD prions at early stage post-inoculation

PrP$^{0/0}$ mice were inoculated intracranially with vCJD prions (20 µl 20%, as in Figure 1) and euthanized in triplicates from day 2 to day 63. PrP^{res} was purified using the Bio-Rad TsSeE detection kit (see methods section). A brain equivalent of 10 mg was loaded on the gels. Serial dilutions of vCJD PrP^{res} present in the brains of terminally sick tg650 mice were loaded as positive control. Detection was made with Sha31 anti-PrP antibody.

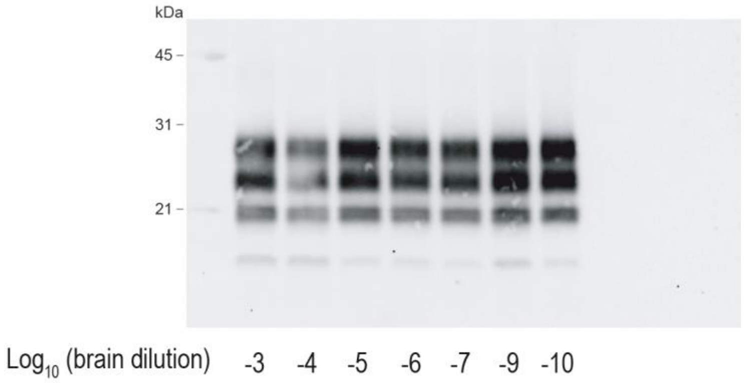

Supplementary Figure 4. PrP^{res} amount in mb-PMCA products generated with different concentrations of 127S prion seed

Brain homogenate from tg338 mice infected with 127S prions was serially diluted in tg338 healthy brain lysate as indicated. Each dilution served as seed for a single round of mb-PMCA. All PMCA products were digested with PK before Western blot using Sha31 anti-PrP antibody.

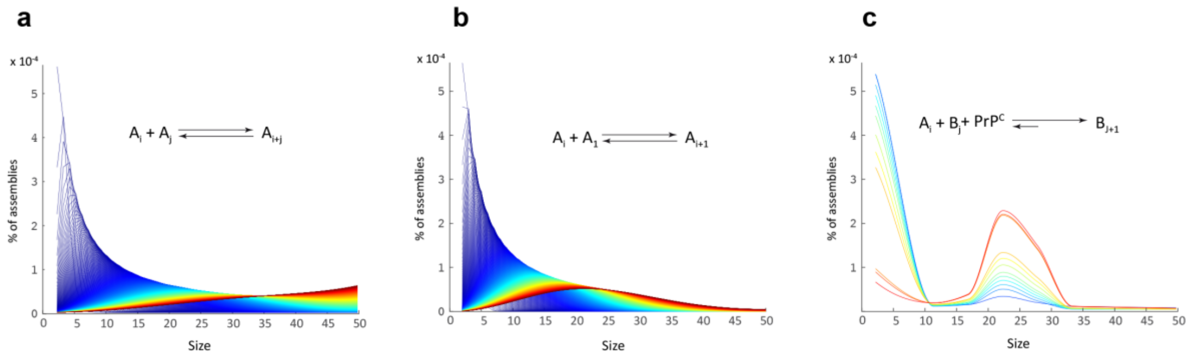

Supplementary Figure 5. Simulation of the size distribution evolution of PrP^{Sc} assemblies according to different molecular processes

During the coalescence or coagulation of structurally equivalent (i.e. size independency of kinetic constant) polymers or protein assemblies, the size distribution evolution is precisely described through a well-studied process known as Smoluchowski coagulation process (which is similar to Oswald repining). The coagulation could occur either by coalescence between polymeric assemblies or by a specific sub-element transfer through equilibrium displacement. The mathematics of the coagulation by coalescence or sub-element transfer are similar and can be formalized precisely by Smoluchowski master equation describing the evolution of the size distribution as function of time[1,2]. Indeed, if one considers the existence of structurally identic set of assemblies differing uniquely by size: A_i and A_j with $i \geq j$, their time dependency dynamic is described as a discreet system by the two-couple ordinary differential equations (ODE):

$$A_i + A_j \rightleftharpoons A_{i+j} \qquad i \geq j;$$

$$\frac{d(A_i)}{dt} = -k_1(A_i)(A_j) + k_2(A_{i+j}) \qquad (1)$$

$$\frac{d(A_{i+j})}{dt} = k_1(A_i)(A_j) - k_2(A_{i+j}) \qquad (2)$$

The equations 1 and 2 can be used to simulate the dynamic of size distribution when the concentration of either one type or a group of assemblies increases. As shown in (**a**), when the concentration of a subgroup of assemblies increases, the size distribution is continuous. Similar observation can be made when assemblies coagulation occurs through exchange of a specific subtype of object, here A_1 (**b**). Thus, coagulation and coalescence phenomenon cannot explain the bimodal kinetic behavior observed during the quiescent phase. Only the existence of structurally distinct set of assemblies with distinct kinetic parameter could support the bimodal behavior (**c**).

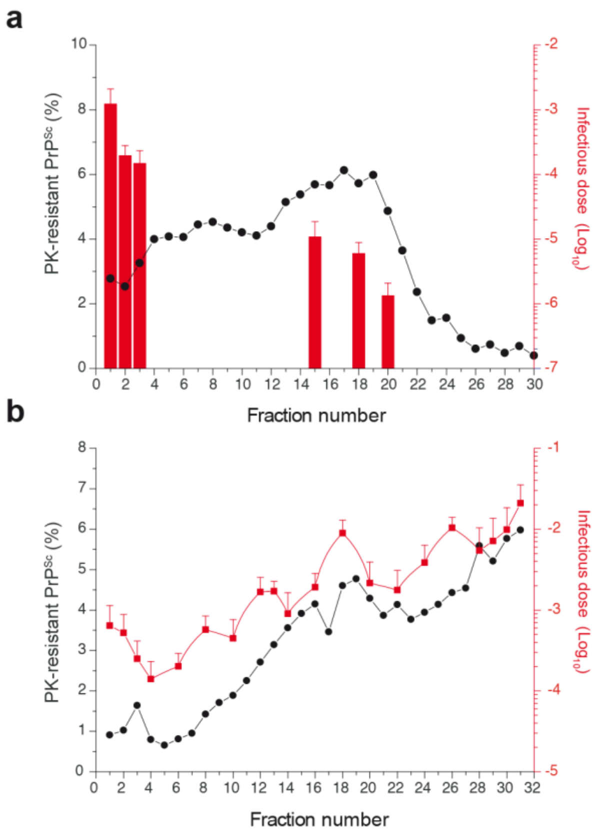

Supplementary Figure 6. PK-resistant PrP^{Sc} and infectivity sedimentation profiles of 139A and vCJD prion strains

Brain homogenates from (**a**) tga20 mice infected with 139A prions and (**b**) tg650 mice infected with vCJD prions were solubilized and SV-fractionated. The collected fractions were analyzed for PK-resistant PrP^{Sc} content (black line) and for infectivity (red bars or line) with an incubation time bioassay in reporter tga20 and tg650 mice. The mean survival time values of these mice were reported to standard dose-response curves ([3,4] and unpublished) to determine relative infectious dose values. A relative infectious dose of 0 corresponds to animals inoculated with 2 mg of infectious brain tissue.

Supplementary Note: Mathematical modelling

1 Reaction scheme

We consider two different kinds of oligomers: on the one hand, A_i, of size $2i$, are formed by the aggregation of i $SuPrP^A$ formed of two monomers, and denoted $A_1 = SuPrP^A$. On the second hand, oligomers B_i, of size $3i$, able to aggregate by $SuPrP^B$ addition, where $SuPrP^B$ is another SuPrP formed of three monomers. However, $SuPrP^A = A_1$ may react with monomers to give rise to $SuPrP^B$. Let us also note that the size of $SuPrP^A$ and $SuPrP^B$, respectively formed of two and three monomers in the following model, is somewhat arbitrary: all we know is that this is their order of magnitude, in coherence with reference 4.

Let us recall the reaction scheme explained and justified in the Main Text.

1. The oligomers A_i follow a classical polymerization/depolymerization chain reaction, by $SuPrP^A = A_1-$ addition (Eq. (1) in the Main Text):

$$A_1 + A_i \underset{k_{dep,a}^{i+1}}{\overset{k_{on,a}^i}{\rightleftharpoons}} A_{i+1}, \qquad 1 \leq i \leq n-1.$$

2. The oligomers B_i follow a classical polymerization/depolymerization chain reaction, by B_1- addition (Eq. (2) in the Main Text):

$$B_1 + B_i \underset{k_{dep}^{i+1}}{\overset{k_{on}^i}{\rightleftharpoons}} B_{i+1}, \qquad 1 \leq i \leq n-1.$$

3. A_1 and B_1 can form a complex C in a reversible way (Eq. (3) in the Main Text):

$$A_1 + B_1 \underset{k_C^-}{\overset{k_C^+}{\rightleftharpoons}} C.$$

4. The complex C can then react with the monomer M to form two B_1 (Eq. (4) in the Main Text):

$$M + C \overset{k_+}{\longrightarrow} 2B_1.$$

We translate this reaction scheme into the following system of differential equations, denoting a_1, a_i, b_1, b_i and c respectively the concentrations of A_1, A_i, B_1, B_i and C:

$$\frac{dm}{dt} = -k_+ m(t)c(t), \qquad m(0) = m^0, \tag{1.1}$$

$$\frac{dc}{dt} = k_C^+ a_1(t)b_1(t) - k_C^- c(t) - k_+ m(t)c(t), \qquad c(0) = c^0, \tag{1.2}$$

$$\frac{da_1}{dt} = -k_C^+ a_1(t)b_1(t) + k_C^- c(t) - J_1^a - \sum_{i=1}^{n-1} J_i^a, \qquad a_1(0) = a_1^0, \tag{1.3}$$

$$\frac{db_1}{dt} = -k_C^+ a_1(t)b_1(t) + k_C^- c(t) + 2k_+ m(t)c(t) - J_1 - \sum_{i=1}^{n-1} J_i, \tag{1.4}$$

$$\frac{db_i}{dt} = J_{i-1} - J_i, \qquad \frac{da_i}{dt} = J_{i-1}^a - J_i^a, \qquad 2 \leq i \leq n-1, \tag{1.5}$$

$$\frac{db_n}{dt} = J_{n-1}, \qquad \frac{da_n}{dt} = J_{n-1}^a, \qquad b_i(0) = b_i^0, \quad a_i(0) = a_i^0, \quad 1 \leq i \leq n, \tag{1.6}$$

where J_i and J_i^a are the net rate at which a polymer of size $2i$ (resp. $3i$) grows into a polymer of size $3(i+1)$ (resp. $2(i+1)$), hence:

$$J_i = k_{on}^i b_1 b_i - k_{dep}^{i+1} b_{i+1}, \qquad J_i^a = k_{on,a}^i a_1 a_i - k_{dep,a}^{i+1} a_{i+1}.$$

2 Analysis and calibration of the model

Due to the fact that $i_A < 5$, as A_i assemblies are eluded in the first Sedimentation Velocity (S.V) fractions, we first decide to neglect the oligomers A_i with $i > 1$ for the sake of model simplicity. This implies that we keep only in the system $SuPrP^A$ and neglect the quantities a_i for $i \geq 2$, so that $J_i^a = 0$ for all i in the previous system.

The parameters to estimate are then: m^0, c^0, a_1^0, b_i^0, k_+, k_C^+, k_C^-, k_{on}^i and k_{dep}^{i+1} - total of $2(n-1) + n + 6 = 3n + 4$ parameters if there are n different sizes of polymers.

We can however use the properties of the model to determine part of the parameters.

2.1 Interpretation of the S.V data

The data obtained by S.V are interpreted as a dilatation of a size distribution density, normalized at 100%, so that if u_i denotes the concentration of polymers formed of i monomers, the data represent $\frac{iu_i}{\sum\limits_k ku_k}$.

In the absence of an exactly reliable relation between the fraction number and the sizes of the oligomers, we assume (a choice which is qualitatively acceptable) that if $O_j(t)$ denotes the proportion of the fraction number j at time t, it measures the proportion of polymerized mass present in oligomers containing roughly $j-$ monomers.

In the following, we denote the total polymerized mass as

$$\mathcal{M}(t) := 2a_1(t) + 3b_1(t) + 5c(t) + 3\sum_{i=2}^{n} ib_i(t).$$

We thus interpret the fraction number measured as follows:

$$\frac{2a_1(t) + 3b_1(t) + 5c(t)}{\mathcal{M}(t)} \approx \sum_{j=1}^{5} O_j(t) := \mathcal{O}_1(t), \qquad \frac{3ib_i(t)}{\mathcal{M}(t)} \approx \sum_{j=3i}^{3i+2} O_j(t) := \mathcal{O}_i(t), \quad i \geq 2,$$

and we use in the following the quantities $\mathcal{O}_i(t)$, measured at several time points, to compare the model with the experimental data. We have a maximal fraction number equal to 30, so that we define $\mathcal{O}_j$ for $j \leq 9$ and add the value of O_{30} to compute $\mathcal{O}_9$ in the above definition.

Let us recall here that the size of three monomers for suPrP-B constitutes itself an approximation, so that the fit of our model to the experimental data is meant as a qualitative insight.

2.2 Conserved quantities

The system has two conserved quantities: first, the total mass:

$$\frac{d}{dt}\Big(m + 2a_1 + 5c + 3\sum_{i=1}^{n} ib_i\Big) = 0 = \frac{d}{dt}\Big(m(t) + \mathcal{M}(t)\Big),$$

and second, what can be viewed as the excess of monomers which will not be consumed to form $suPrP^B$:

$$\frac{d}{dt}\Big(m - a_1 - c\Big) = 0.$$

We denote these conserved quantities respectively $\mathcal{M}_{tot} = m^0 + 2a_1^0 + 5c^0 + 3\sum\limits_{i=1}^{n} ib_i^0$ and $\rho^0 = m^0 - a_1^0 - c^0$. These two quantities depend on the parameters to be estimated.

A quantity directly measured experimentally is the so-called *centroid*, defined as the average size:

$$centroid(t) := \frac{\mathcal{M}_2(t)}{\mathcal{M}(t)} = \frac{4a_1(t) + 9b_1(t) + 9\sum\limits_{i=1}^{9} i^2 b_i(t)}{2a_1(t) + 3b_1(t) + 3\sum\limits_{i=1}^{9} ib_i(t)} \approx \frac{\sum\limits_{j=1}^{30} jO_j(t)}{\sum\limits_{j=1}^{30} O_j(t)}.$$

2.3 Asymptotic and initial behaviour of the model

We consider that at the final time measurement, an equilibrium has been reached, that we denote with $^\infty$ superscripts. The equilibrium fulfills the following equations:

$$\begin{cases} -k_+ m^\infty c^\infty &= 0, \\ k_C^+ a_1^\infty b_1^\infty - k_C^- c^\infty &= 0, \\ J_1^\infty = \ldots = J_9^\infty &= 0. \end{cases} \tag{2.1}$$

Asymptotically, if the monomers are in excess, the system converges towards the following state:

$$c^\infty = a_1^\infty = 0, \qquad m^\infty = \rho^0, \qquad b_i^\infty = \frac{k_{on}^{i-1}}{k_{dep}^i} b_1^\infty b_{i-1}^\infty, \quad i \geq 2.$$

The last equality allows us to define recursively b_i^∞ from b_1^∞, and b_1^∞ is given by the following mass equality:

$$\mathcal{M}^\infty = 3 \sum_{i=1}^{9} i b_i^\infty.$$

Since $\mathcal{O}_1^\infty = \frac{3b_1^\infty}{\mathcal{M}^\infty}$, we have

$$\frac{k_{on}^{i-1}}{k_{dep}^i} \mathcal{M}^\infty = \frac{3b_i^\infty}{b_{i-1}^\infty \mathcal{O}_1^\infty} = 3 \frac{\mathcal{O}_i^\infty}{\mathcal{O}_{i-1}^\infty \mathcal{O}_1^\infty} \frac{i-1}{i}$$

which can be experimentally measured: this gives us $n-1$ relations, thus we now have $2n+5$ parameters to estimate (here $n = 9$).

We also assume that initially, before adding monomers, the system was in equilibrium, which means:

$$k_C^- c^0 = k_C^+ a_1^0 b_1^0, \qquad \frac{b_i^0}{b_{i-1}^0 b_1^0} = \frac{k_{on}^{i-1}}{k_{dep}^i} = \frac{\mathcal{O}_i^0}{b_1^0 \mathcal{O}_{i-1}^0} \frac{i-1}{i}, \qquad i \geq 3,$$

so that we have $n-1$ new relations, and the number of parameters to estimate is reduced to $n+6$.

2.4 Numerical simulations

We run the simulations with Matlab, and used the ode solver ode45. The parameters which were not given by the analysis have been adjusted qualitatively. The time scale is in hours, the concentrations are in arbitrary units.

k_{on}^1	k_{on}^2	k_{on}^3	k_{on}^4	k_{on}^5	k_{on}^6	k_{on}^7	k_{on}^8
8.4	52.6	166	4.4	0.35	5.4	5.6	4.8
k_{dep}^2	k_{dep}^3	k_{dep}^4	k_{dep}^5	k_{dep}^6	k_{dep}^7	k_{dep}^8	k_{dep}^9
100	100	100	25	25	25	25	25
$b_2(0)$	$b_3(0)$	$b_4(0)$	$b_5(0)$	$b_6(0)$	$b_7(0)$	$b_8(0)$	$b_9(0)$
0.42	0.32	0.65	0.27	0.1	0.07	0.046	0.03
k_+^C	k_-^C	k_+	$m(0)$	$c(0)$	$a_1(0)$	$b_1(0)$	
0.15	3000	5	300	7.10^{-4}	38.2	0.39	

Supplementary References

1 Hassan, M. K. & Hassan, M. Z. Condensation-driven aggregation in one dimension. *Phys Rev E Stat Nonlin Soft Matter Phys* **77**, 061404, doi:10.1103/PhysRevE.77.061404 (2008).

2 Smoluchowski, M. Molekular-kinetische Theorie der Opaleszenz von Gasen im kritischen Zustande, sowie einiger verwandter Erscheinungen. *Annalen der Physik* **330**, 205-226 (1908).

3 Tixador, P. *et al.* The physical relationship between infectivity and prion protein aggregates is strain-dependent. *PLoS Pathog* **6**, e1000859, doi:10.1371/journal.ppat.1000859 (2010).

4 Igel-Egalon, A. *et al.* Reversible unfolding of infectious prion assemblies reveals the existence of an oligomeric elementary brick. *PLoS Pathog* **13**, e1006557, doi:10.1371/journal.ppat.1006557 (2017).
