# Supplementary material for: Early stage prion assembly involves two subpopulations with different quaternary structures and a secondary templating pathway
Source: Commun Biol. 2019 Oct 4;2:363. doi: 10.1038/s42003-019-0608-y (PMC6778151; doi:10.1038/s42003-019-0608-y)
Supplement: Supplementary file 2 — Reporting Summary [file 42003_2019_608_MOESM2_ESM.pdf]

nature research

Corresponding author(s): Vincent Beringue; Human Rezaei

Last updated by author(s): Sep 3, 2019

Reporting Summary

Nature Research wishes to improve the reproducibility of the work that we publish. This form provides structure for consistency and transparency in reporting. For further information on Nature Research policies, see Authors & Referees and the Editorial Policy Checklist.

Statistics

For all statistical analyses, confirm that the following items are present in the figure legend, table legend, main text, or Methods section.

n/a	Confirmed	
☐	☒	The exact sample size (*n*) for each experimental group/condition, given as a discrete number and unit of measurement
☐	☒	A statement on whether measurements were taken from distinct samples or whether the same sample was measured repeatedly
☒	☐	The statistical test(s) used AND whether they are one- or two-sided<br>*Only common tests should be described solely by name; describe more complex techniques in the Methods section.*
☒	☐	A description of all covariates tested
☒	☐	A description of any assumptions or corrections, such as tests of normality and adjustment for multiple comparisons
☒	☐	A full description of the statistical parameters including central tendency (e.g. means) or other basic estimates (e.g. regression coefficient) AND variation (e.g. standard deviation) or associated estimates of uncertainty (e.g. confidence intervals)
☒	☐	For null hypothesis testing, the test statistic (e.g. *F*, *t*, *r*) with confidence intervals, effect sizes, degrees of freedom and *P* value noted<br>*Give P values as exact values whenever suitable.*
☒	☐	For Bayesian analysis, information on the choice of priors and Markov chain Monte Carlo settings
☒	☐	For hierarchical and complex designs, identification of the appropriate level for tests and full reporting of outcomes
☒	☐	Estimates of effect sizes (e.g. Cohen's *d*, Pearson's *r*), indicating how they were calculated

Our web collection on statistics for biologists contains articles on many of the points above.

Software and code

Policy information about availability of computer code

Data collection	Immunoblot chemiluminescence: Chemidoc digital imager (Bio-Rad)
Data analysis	Immunoblot: ImageLab software (Bio-Rad)<br>Graphs: Origin 8 (OriginLab Corporation)

For manuscripts utilizing custom algorithms or software that are central to the research but not yet described in published literature, software must be made available to editors/reviewers. We strongly encourage code deposition in a community repository (e.g. GitHub). See the Nature Research guidelines for submitting code & software for further information.

Data

Policy information about availability of data

All manuscripts must include a data availability statement. This statement should provide the following information, where applicable:

- Accession codes, unique identifiers, or web links for publicly available datasets
- A list of figures that have associated raw data
- A description of any restrictions on data availability

The SV and SEC raw data set have been deposited to the DRYAD depository with the identifier DOI: doi:10.5061/dryad.f28b4m6. All other data are available from the corresponding authors on request.

Field-specific reporting

Please select the one below that is the best fit for your research. If you are not sure, read the appropriate sections before making your selection.

☒ Life sciences ☐ Behavioural & social sciences ☐ Ecological, evolutionary & environmental sciences

For a reference copy of the document with all sections, see nature.com/documents/nr-reporting-summary-flat.pdf

Life sciences study design

All studies must disclose on these points even when the disclosure is negative.

Sample size	No statistical methods were used to pre-determine sample sizes. Those have been established according to our previous publications (Tixador et al, PLoS Pathogens 2010; Laferreriere et al., PLoS Pathogens 2013; Moudjou et al, mBio 2013; Igel-Egalon et al, PLoS Pathogens 2017)
Data exclusions	No data were excluded from the analyses
Replication	All experiments mentionned in the study were replicated as mentioned in the manuscript. All attempts at replication were sucessfull
Randomization	SV-fractionation and PMCA procedures were randomized with control samples
Blinding	Blinding was not relevant to our study as data collection and analysis did not involve any subjective measurement.

Reporting for specific materials, systems and methods

We require information from authors about some types of materials, experimental systems and methods used in many studies. Here, indicate whether each material, system or method listed is relevant to your study. If you are not sure if a list item applies to your research, read the appropriate section before selecting a response.

Materials & experimental systems

n/a	Involved in the study	
☐	☒	Antibodies
☒	☐	Eukaryotic cell lines
☒	☐	Palaeontology
☐	☒	Animals and other organisms
☒	☐	Human research participants
☒	☐	Clinical data

Methods

n/a	Involved in the study	
☒	☐	ChIP-seq
☒	☐	Flow cytometry
☒	☐	MRI-based neuroimaging

Antibodies

Antibodies used	Sha31 was used as anti-PrP antibody. This antibody is described in Feraudet et al., 2005 (ref). Sha31 is widely used in the prion field to detect PrP isoforms by western blot since more than 15 years.<br>Sha31 is sold by Caiman chemical, catalogue No. 11866<br>It was used at 0.1 µg/ml final concentration
Validation	See manufacturer's webiste: https://www.caymanchem.com/product/11866

Animals and other organisms

Policy information about studies involving animals; ARRIVE guidelines recommended for reporting animal research

Laboratory animals	transgenic mouse lines expressing human PrP (tg650), ovine PrP (tg338), hamster PrP (tg7) or no PrP (Zurich 1) were used. Eight-week-old female were used.
Wild animals	No wild-type animals were used
Field-collected samples	No-field collected samples were used
Ethics oversight	Animal experiments were conducted in strict compliance with ECC and EU directives 86/009 and 2010/63 and were approved by the local ethics committee of the author's institution (Comethea; permit numbers 12/034 and 15/045).

Note that full information on the approval of the study protocol must also be provided in the manuscript.
